# Chronische Otitis media mesotympanalis mit atypischem Verlauf: Eine seltene Differenzialdiagnose

**DOI:** 10.1007/s00106-026-01783-9

**Published:** 2026-06-17

**Authors:** R. Baldissera, C. Schmit, G. Widmann, J. Schmutzhard, B. Hofauer

**Affiliations:** 1 Universitätsklinik für Hals‑, Nasen‑, Ohrenheilkunde, Medizinische Universität Innsbruck, Anichstr. 35, 6020 Innsbruck, Österreich; 2 Universitätsklinik für Radiologie, Medizinische Universität Innsbruck, Innsbruck, Österreich

## Anamnese

Eine 49-jährige Patientin stellte sich mit seit Jahren bestehenden Ohrenbeschwerden, in der Hals-Nasen-Ohren(HNO)-Klinik vor.

Die ursprünglich aus dem Nahen Osten stammende Patientin berichtete anamnestisch über rezidivierend auftretende Otorrhö sowie eine subjektive Hörminderung. Sonstige otologische Beschwerden wie Tinnitus, Schwindel oder Otalgie wurden verneint.

Bis auf eine vorbekannte und medikamentös eingestellte arterielle Hypertonie bestanden zum Zeitpunkt der Erstvorstellung keinerlei relevante Vorerkrankungen.

Die Zuweisung in die HNO-Klinik erfolgte zur präoperativen Abklärung und Planung einer Tympanoplastik.

## Klinische Untersuchung

Ohrmikroskopisch zeigten sich rechtsseitig regelrechte anatomische Verhältnisse. Linksseitig fanden sich 2 getrennte Trommelfellperforationen – eine auf die vorderen beiden Quadranten begrenzte und eine weitere im unteren hinteren Quadranten. Beide Perforationen wiesen keine Zeichen einer akuten Superinfektion auf.

**Abb. 1:**
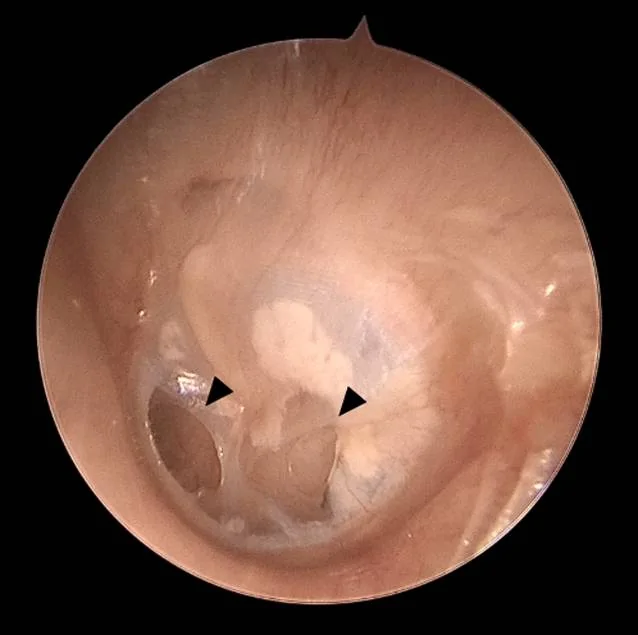
Endoskopische Sicht auf das linke Trommelfell: multiple Trommelfellperforationen (*Pfeilspitzen*) und diffuse Sklerosierung der übrigen Trommelfellanteile

Der Anulus fibrosus war intakt, die Paukenschleimhaut erschien unauffällig. Das restliche Trommelfell erschien teils sklerosiert und verdickt. Der übrige HNO-Status war unauffällig (Abb. 1).

**Abb. 2:**
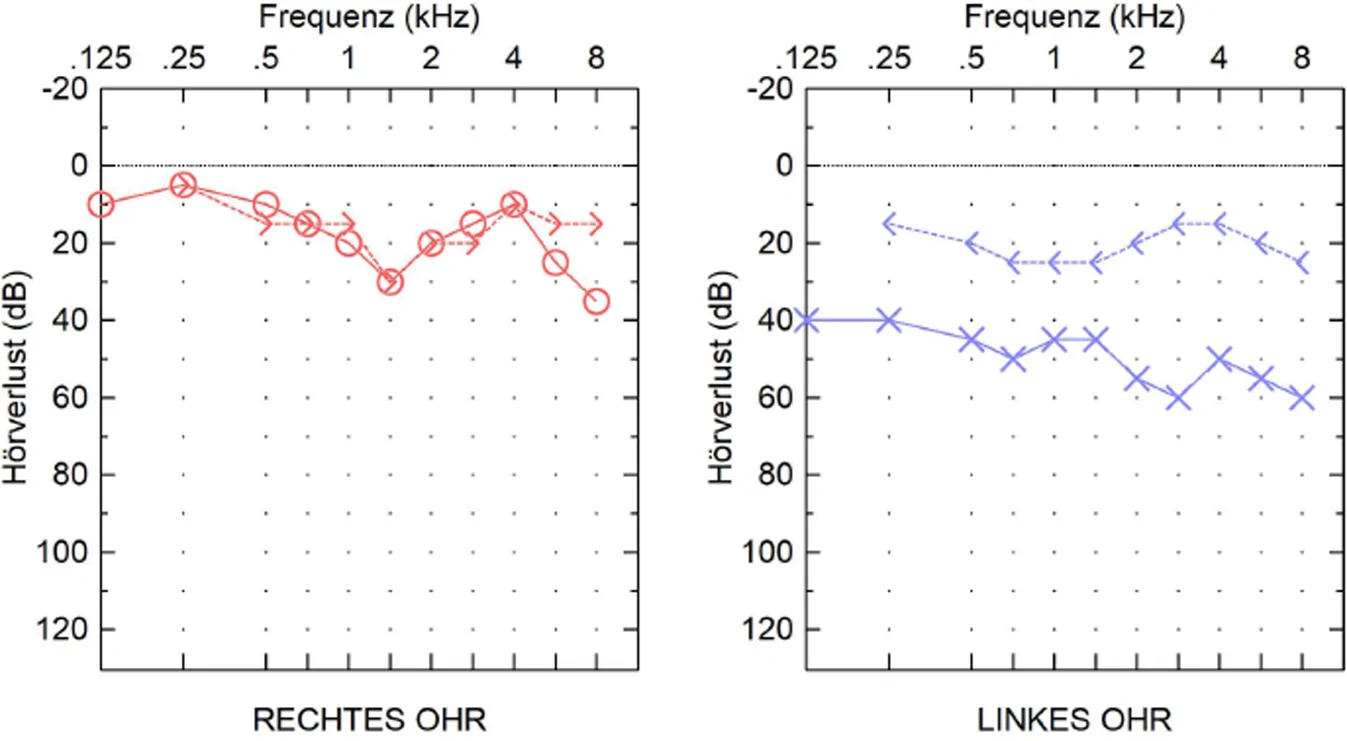
Tonaudiogramm: Linksseitig (*blau*) zeigt sich eine ausgeprägte Schallleitungsschwerhörigkeit mit Schalleitungskomponente von bis zu 45 dB

Im Tonaudiogramm zeigten sich rechtsseitig ein altersentsprechender, normakustischer Befund und linksseitig eine ausgeprägte Schallleitungsschwerhörigkeit mit einem maximalen „air-bone gap“ von bis zu 45 dB (Abb. 2).

## Diagnostik und weiteres Prozedere

In der präoperativ durchgeführten Computertomographie (CT) des Felsenbeins zeigten sich keine richtungsweisenden pathologischen Befunde.

**Abb. 3:**
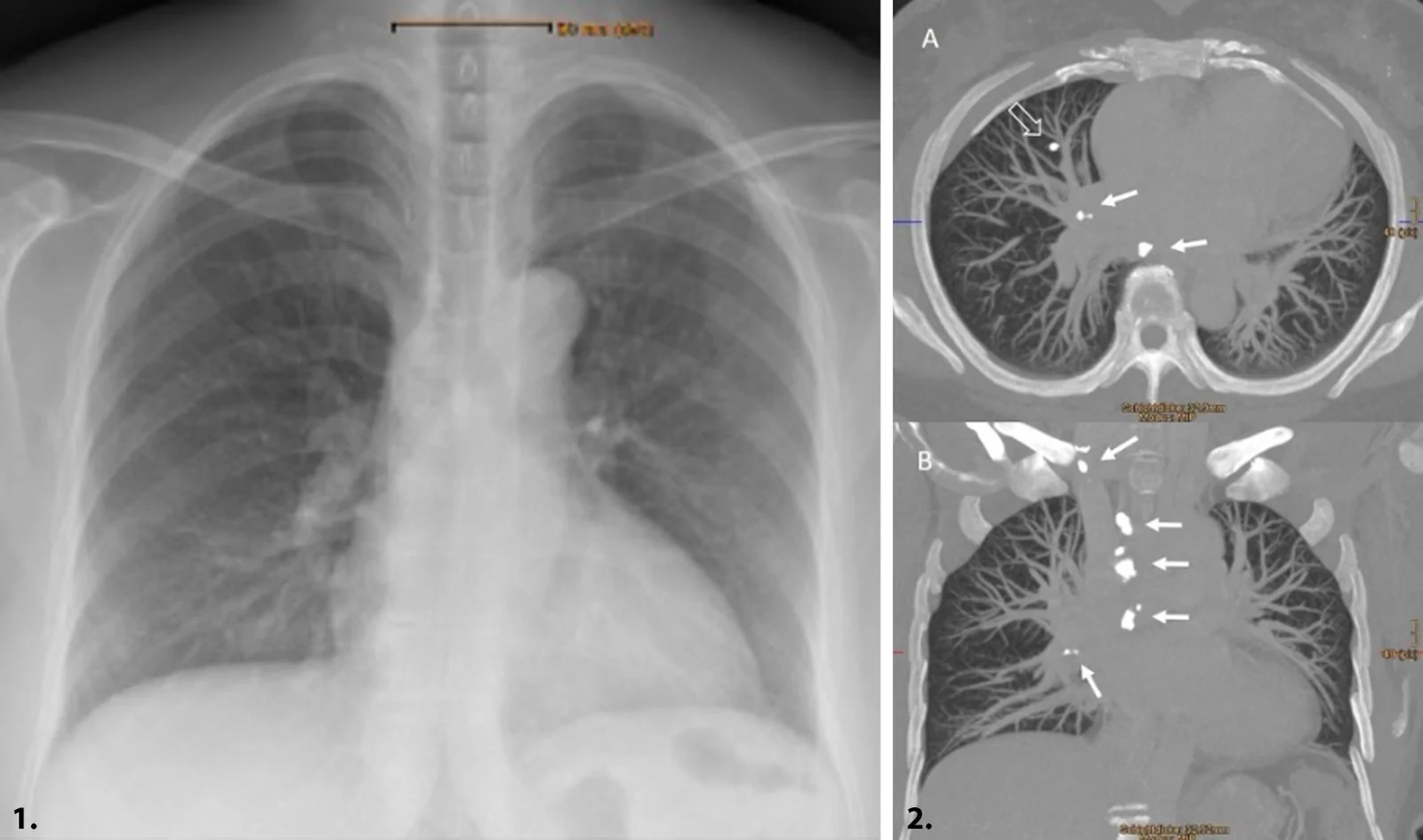
**1** Thoraxröntgen: multiple verkalkte Lymphknoten mediastinal, keine sonstige Akutpathologie darstellbar. **2** Computertomographie des Thorax: Die axiale rekonstruierte Maximum-intensity-Projektion zeigt ein posttuberkulöses Granulom im Mittellappen (**2A**, *offener Pfeil*) und verkalkte rechte Hilus- und Infrakarinallymphknoten (**2A**, *Pfeile*). **2B** Die koronare rekonstruierte Maximum-intensity-Projektion zeigt multiple verkalkte rechte Mediastinal- und rechte Hiluslymphknoten

In dem im Rahmen der anästhesiologischen Freigabe veranlassten Thoraxröntgen zeigten sich mehrere verkalkte mediastinale Lymphknoten. Zur weiterführenden Abklärung erfolgte eine ergänzende Low-dose-CT-Thorax-Untersuchung (Abb. 3).

Entsprechend der erhobenen Befunde wurde eine weiterführende differenzialdiagnostische Abklärung eingeleitet.

## Therapie und Verlauf

Aufgrund des klinischen und bildgebenden Befundbildes wurde eine differenzialdiagnostische Abklärung hinsichtlich einer tuberkulösen Genese veranlasst. Die mikrobiologische Untersuchung eines aus dem Bereich der Trommelfellperforationen gewonnenen Abstrichs ergab keinen spezifischen Erregernachweis.

Zur weiterführenden Diagnostik erfolgte die Zuweisung in die infektiologische Ambulanz. Dort ergab sich bei positivem Interferon-gamma-Release-Assay (Quantiferon-Test), dem Nachweis multipler verkalkter pulmonaler Herde in der CT und derzeit fehlender B‑Symptomatik der begründete Verdacht auf eine inaktive Tuberkuloseinfektion. Eine Sputumdiagnostik war bei erschwerter Compliance der Patientin nicht möglich. Aufgrund des erhöhten Risikoprofils wurde eine antituberkulöse Kombinationstherapie initiiert. Im Anschluss an die medikamentöse Therapie zeigte die im Rahmen der operativen Trommelfellrekonstruktion durchgeführte histopathologische Untersuchung der Paukenschleimhaut keine Hinweise auf eine persistierende granulomatöse Entzündung. Der postoperative Verlauf zeigte sich komplikationslos.

## Wie lautet Ihre Diagnose?

**Diagnose:** V. a. tuberkulöse chronische Otitis media mesotympanalis (COMM)

## Diskussion

Der Begriff der chronischen Otitis media dient als Überbegriff für eine Vielzahl von unterschiedlichen Erkrankungen des Mittelohrs, die durch eine persistierende Trommelfellperforation als gemeinsames Merkmal gekennzeichnet sind. Dabei stellt das wichtigste klinische Merkmal der mesotympanalen Form eine zentrale Trommelfellperforation mit erhaltenem Anulus fibrosus dar [1].

Mit einer relativen Häufigkeit von 0,04–0,9 % stellt die tuberkulöse Otitis media (TOM) eine seltene ätiologische Form der chronischen Mittelohrentzündung dar. Die Infektion kann hämatogen, über die Tuba auditiva oder durch direkte Inokulation bei bestehender Trommelfellperforation erfolgen [1].

Das klinische Erscheinungsbild der TOM ist ausgesprochen variabel. In der Literatur werden klassische klinische Verläufe mit schmerzloser Otorrhö und Hörminderung, multiplen Trommelfellperforationen sowie einhergehender Fazialisparese beschrieben. Des Weiteren kann sich die TOM ebenso in Form einer Mastoiditis manifestieren, sowie mit postauralen Abszessen oder Fistulierungen einhergehen [1, 2]. In Analogie zum vorliegenden Fall sind jedoch auch Verläufe mit makroskopisch unauffälliger Paukenschleimhaut dokumentiert [3].

Die zur weiterführenden strukturellen Abklärung eingesetzte Felsenbein-CT liefert oft nur unspezifische Merkmale. Gut belüftete Mastoidzellen, eine Weichteilvermehrung in den Mittelohrräumen oder resultierende Knochenarrosion stellen mögliche radiologische Diagnosekriterien dar [2, 4].

Die klinische Suspizion kann durch weitere flankierende diagnostische Maßnahmen, wie z. B. einen positiven Quantiferon-Test, bildgebende pulmonale Auffälligkeiten oder auffällige Sputumdiagnostik, gestützt werden.

Wie auch im beschriebenen Fall gestaltet sich der mikroskopische Nachweis säurefester Stäbchen im Ohrsekret oft frustran, sodass oft erst eine histopathologische Untersuchung von aus dem Mittelohr entnommenem Granulationsgewebe eine Diagnose erlaubt [5].

Die antituberkulöse Kombinationstherapie stellt den Grundpfeiler der Therapie dar und wird auch in der Behandlung einer, wie im vorliegenden Fall, inaktiven bzw. latenten Tuberkulose eingesetzt [2]. Obwohl in der Literatur vereinzelt spontane Remissionen einer tuberkulösen COMM unter alleiniger tuberkulostatischer Therapie beschrieben sind, gilt die chirurgische Sanierung hinsichtlich der funktionellen Rehabilitation weiterhin als überlegen [6].

Chirurgische Interventionen können bereits im Rahmen der für die Diagnosestellung häufig erforderlichen Probengewinnung notwendig sein. Weitere Indikationen für eine chirurgische Sanierung stellen subperiostale Abszesse, das Ausräumen von Knochensequestern und Entlastungen des N. facialis dar [7].

Bedingt durch ihre Seltenheit und das klinisch variable Erscheinungsbild stellt die TOM weiterhin eine diagnostische und therapeutische Herausforderung dar [5]. Insbesondere bei Patient:innen aus Tuberkuloseendemiegebieten sollte bei chronischer Otorrhö, multiplen Trommelfellperforationen oder ausgeprägter Schallleitungsschwerhörigkeit frühzeitig eine differenzialdiagnostische Abklärung hinsichtlich einer tuberkulösen Genese erfolgen. Die diagnostische Herausforderung liegt dabei v. a. in der häufig unspezifischen klinischen Präsentation und im oftmals ausbleibenden direkten mikrobiologischen Erregernachweis.

Obwohl im beschriebenen Fall ein direkter kausaler Zusammenhang zwischen der chronischen Otitis media und der, am ehesten ausgeheilten, Tuberkulose histopathologisch nicht gesichert werden konnte, liegt in Zusammenschau der klinischen, bildgebenden und laborchemischen Befunde eine entsprechende Ätiopathogenese nahe.

## Fazit für die Praxis

- Bei persistierender Otorrhö, multiplen Trommelfellperforationen und ausgeprägter Schallleitungsschwerhörigkeit sollte differenzialdiagnostisch auch an eine tuberkulöse Otitis media als seltene extrapulmonale Manifestation gedacht werden.
- Ein mikroskopischer Erregernachweis aus dem Ohrsekret gestaltet sich meist frustran.
- Die histopathologische Untersuchung von Granulationsgewebe bleibt entscheidend für die Diagnosesicherung.
- Die antituberkulöse Kombinationstherapie ist zentraler Bestandteil der Behandlung.
- Flankierende chirurgische Maßnahmen dienen neben der Diagnosestellung und Komplikationsbehandlung insbesondere auch der funktionellen Rehabilitation und Hörverbesserung.

## Data Availability

Alle dieser Arbeit zugrunde liegenden Daten sind in diesem Artikel enthalten.
